# Unveiling etiology and mortality risks in community-acquired pneumonia: A machine learning approach

**DOI:** 10.17305/bb.2025.12378

**Published:** 2025-06-18

**Authors:** Alaa Ali, Ahmad R Alsayed, Nesrin Seder, Yazun Jarrar, Raed H Altabanjeh, Mamoon Zihlif, Osama Abu Ata, Anas Samara, Malek Zihlif

**Affiliations:** 1 Department of Clinical Pharmacy and Therapeutics, Applied Science Private University, Amman, Jordan; 2 Department of Pharmaceutical Chemistry and Pharmacognosy, Applied Science Private University, Amman, Jordan; 3 Department of Basic Medical Sciences, Faculty of Medicine, Al-Balqa Applied University, Al-Salt, Jordan; 4 Department of Internal Medicine, Section of Pulmonary, Islamic Hospital, Amman, Jordan; 5 Department of Internal Medicine, Section of Infectious Diseases, Islamic Hospital, Amman, Jordan; 6 Department of Software Engineering, Bethlehem University, Bethlehem, Palestine; 7 Department of Pharmacology, School of Medicine, The University of Jordan, Amman, Jordan

**Keywords:** Community-acquired pneumonia, CAP, machine learning, ML, mortality prediction, risk assessment, clinical predictors, SHAP analysis, logistic regression

## Abstract

Community-acquired pneumonia (CAP) is associated with high mortality, and accurate diagnosis and risk prediction are essential for improving patient outcomes. Traditional diagnostic methods have limitations, prompting the use of machine learning (ML) to enhance diagnostic precision and treatment strategies. This study aims to develop ML models to predict CAP etiology and mortality using clinical data to enable early intervention. A retrospective cohort study was conducted on 251 adult CAP patients admitted to two Jordanian hospitals between March 2021 and February 2024. Various clinical data were analyzed using ML techniques, including linear regression, random forest, Shapley additive explanations (SHAP), lasso regression, mutual information analysis, logistic regression, and correlation analysis. Key predictors of CAP survival included zinc, vitamin C, enoxaparin, and insulin bolus. Mutual information analysis identified neutrophils, alanine transaminase, mean corpuscular volume, hemoglobin, and platelets as significant mortality predictors, while lasso regression highlighted meropenem, arterial blood gases, PCO_2_, and platelet count. Logistic regression confirmed intensive care unit (ICU) stay, pH, pulmonary severity index, white blood cell (WBC) count, and bicarbonate levels as crucial variables. Interestingly, lymphocyte count emerged as the strongest predictor of bacterial CAP, conflicting with established knowledge that associates neutrophils with bacterial infections. However, findings related to HCO_3_, blood urea nitrogen, and WBC levels were consistent with clinical expectations. SHAP analysis highlighted basophils and fever as key predictors. Further investigation is needed to resolve conflicting findings and optimize predictive models. ML offers promising applications for CAP prognosis but requires refinement to address discrepancies and improve reliability in clinical decision-making.

## Introduction

Community-acquired pneumonia (CAP) is a significant public health challenge worldwide, contributing to considerable morbidity and mortality across various age groups [1]. Defined as pneumonia acquired outside of a hospital or healthcare setting, CAP remains a leading cause of death not only in underdeveloped regions but also in developed countries [2]. Effective management of CAP, essential for improving patient outcomes, relies heavily on the accurate and timely identification of its etiology and the assessment of potential mortality risks. Machine learning (ML), a key component of artificial intelligence (AI), has shown promising results in enhancing diagnostic precision, optimizing therapeutic strategies, and predicting clinical outcomes in various medical fields [3]. However, the potential of ML to transform clinical approaches to CAP has yet to be fully realized, particularly in integrating diverse datasets to predict disease etiology and outcomes [4]. This study aims to bridge this gap by focusing on employing ML techniques to predict the causes and mortality associated with CAP. The integration of ML tools into CAP management has the potential to significantly enhance diagnostic accuracy, inform treatment plans, and ultimately improve prognostic outcomes for patients [5]. By exploring these possibilities, this research aims to reinforce the evidence base by reliably correlating clinical data with patient prognosis through sophisticated algorithmic analysis. The successful implementation of this research could revolutionize the management of CAP, leading to more personalized healthcare and better resource distribution in treating this prevalent disease. CAP remains a significant healthcare challenge, ranking globally as a major cause of morbidity and mortality [1]. Each year, CAP leads to numerous hospital admissions, placing a heavy burden on medical staff, especially in high-risk groups such as the elderly and those with immune-compromising conditions [6]. The clinical presentations of CAP vary widely, ranging from mild respiratory symptoms to severe cases requiring intensive care. This variability is largely due to the wide range of pathogens that can cause CAP, including bacteria, viruses, and atypical organisms [7]. Current tools for diagnosing CAP, such as chest X-rays, microbial cultures, and molecular tests, often fail to immediately and accurately identify the causative agent. This limitation complicates the selection of appropriate treatment regimens, ultimately impacting patient outcomes [8]. Additionally, the growing resistance to antimicrobial agents among common respiratory pathogens further complicates CAP management, underscoring the importance of effective, accurately targeted initial empirical therapy [9]. Predicting outcomes for CAP patients also poses challenges, arising from the variability in patient responses to the disease. Factors such as underlying health conditions and age significantly influence outcomes. Current predictive models lack the precision needed to guide critical decisions about treatment intensity or hospitalization [10, 11]. Given these challenges, there is a pressing need for enhanced diagnostic and predictive capabilities in managing CAP. ML presents a promising solution due to its ability to process large and complex datasets, potentially uncovering patterns that improve diagnostic accuracy, personalize treatment, and predict outcomes more effectively [12]. These advancements could significantly improve clinical decision-making, reducing treatment failures and minimizing the health burden associated with CAP. The study of ML applications in predicting the etiology and mortality of CAP is crucial for several reasons. First, enhancing diagnostic accuracy directly impacts treatment efficacy. Accurate early diagnosis allows for timely and targeted treatment, which is critical in reducing disease severity and improving recovery rates. Second, improving mortality prediction enables healthcare providers to make more informed decisions about the level of care required. Patients with a poor prognosis could be prioritized for intensive interventions, potentially improving survival rates. Conversely, reliable predictors of lower risk could help avoid unnecessary hospital admissions, reducing healthcare costs and minimizing the risk of hospital-acquired infections [13]. Furthermore, integrating ML into medical practice addresses the challenge of clinical variability in CAP treatment, aligning with the goals of precision medicine. This not only improves health outcomes but also personalizes patient care, leading to better patient experiences and improved adherence to treatment plans. Lastly, the healthcare industry stands to benefit from improved resource distribution. ML can ensure that staffing, equipment, and medications are available when needed, enhancing the overall efficiency of healthcare delivery systems [14]. This research seeks to apply ML comprehensively within the healthcare sector, demonstrating how technology can intersect with clinical expertise to significantly improve patient outcomes and the operational efficiency of healthcare systems. Despite the extensive use of ML in medical diagnostics, its application in predicting the etiology and mortality of CAP remains underexplored. Most existing studies focus primarily on diagnosis and treatment outcomes but rarely combine ML techniques to predict causal pathogens and associated mortality rates based on large datasets. This gap is particularly significant because timely and accurate determination of CAP etiology and prognosis could significantly enhance treatment strategies and patient outcomes. The intersection of CAP management and ML offers significant potential to improve healthcare outcomes. However, there is a noticeable lack of research integrating these two fields. While the literature comprehensively addresses CAP diagnostic procedures and treatment methods [2], as well as the application of ML in medical diagnostics separately [12], few studies have specifically explored the use of ML to predict CAP etiology and mortality. The current landscape of clinical prediction models for diseases like CAP reveals a critical gap in using predictive modeling to anticipate both the disease etiology and outcomes [15]. While studies have demonstrated the feasibility of using ML to predict outcomes in pneumonia cases [16], there is limited focus on directly correlating these outcomes with causative pathogens—a crucial component for determining appropriate therapeutic approaches [15]. Research in fields like cardiology and oncology has made significant strides in utilizing ML technologies [17]. However, similar advancements in infectious diseases, particularly CAP, remain limited [18]. While some studies have shown the potential of ML in predicting mortality and disease progression in COVID-19 pneumonia cases [19], and in developing models to predict adverse outcomes in CAP [20], further exploration is needed. Efforts have been made to predict the outcome of SARS-CoV-2 pneumonia based on laboratory findings [20], as well as to develop models for predicting the severity and mortality of COVID-19 pneumonia patients [21]. In pneumonia, where up to 50% of cases lack identified causative pathogens [21], ML could offer a promising avenue for predicting outcomes and informing treatment decisions. By integrating comprehensive patient histories, clinical signs, and diagnostic data with predictive modeling techniques, we could significantly enhance the understanding and management of diseases like CAP. The primary aim of this study is to develop, validate, and implement ML models specifically designed to predict the etiology and mortality of CAP. This involves creating models that utilize clinical data, including diverse symptoms and outcomes associated with CAP. Ultimately, the goal is to provide healthcare professionals with advanced, data-driven tools to enhance decision-making processes, leading to more accurate and timely interventions, personalized treatment regimens, and improved patient prognosis.

## Materials and methods

### Study design and participants

This retrospective cohort multicenter study included 251 adult patients from Prince Hamza Hospital and the Islamic Hospital, ensuring diverse patient demographics, clinical variables, and a comprehensive dataset. The participants were adults diagnosed with CAP who were admitted to the participating hospitals between March 10, 2021, and February 15, 2024. This timeframe allowed for a substantial number of cases, enhancing the statistical power and validity of the study. Inclusion criteria consisted of patients with a confirmed diagnosis of CAP, based on chest radiography or computed tomography (CT) scans, if performed, and presenting symptoms consistent with pneumonia, such as cough, fever, sputum production, and dyspnea. Microbiological laboratory results were included when available. Both intensive care unit (ICU) and ward patients were included to represent a range of disease severities. Exclusion criteria included patients with hospital-acquired pneumonia (HAP), patients transferred from outside hospitals more than 48 h after admission, and those with incomplete medical records. Patients with Human Immunodeficiency Virus (HIV)/Acquired Immunodeficiency Syndrome (AIDS) and those receiving long-term immunosuppressive therapy were also excluded to avoid potential confounding effects related to different immune responses. In compliance with laws and regulations governing medical research, the study ensured the privacy and confidentiality of participant data. The Institutional Review Board (IRB) of each clinical site approved the study, with all data de-identified and stored in secure databases to protect patient confidentiality. The study adheres to the Declaration of Helsinki. Ethical approval was obtained from the Applied Science Private University (Jordan), the Islamic Hospital Ethical Committee in Amman, Jordan, and Prince Hamza Hospital in Amman, Jordan (2021-PHA-35, IRB: 101/2021/1053, and 6-11-2021-129, respectively).

### Data collection

The data for the participants was gathered from medical records and electronic databases. Originally recorded in Excel, the dataset contains information about CAP patients, including vital signs, physical and laboratory findings, length of stay (LOS), and in-hospital mortality. This information was sourced from electronic medical records, with additional input from partnerships with hospitals and healthcare providers treating CAP patients. The key elements of the data collection process include:

1. **Demographic information**: Age, gender, and other relevant demographic factors that can influence disease outcomes.
2. **Clinical data**: Detailed records of symptoms, duration of illness, previous health conditions, and clinical findings during physical examinations.
3. **Radiological data**: The radiological data (Chest X-rays, and CT scans) were interpreted by two clinicians, who provided standardized findings such as the presence of pulmonary infiltrates, consolidation, or effusion
4. **Microbiological data**: Results from respiratory sample cultures, blood tests, and other relevant microbiological investigations like the molecular methods (the real-time polymerase chain reaction [PCR]) used to determine the etiology of pneumonia.
5. **Laboratory results**: Complete blood counts, C-reactive protein levels, arterial blood gases, and other relevant laboratory tests performed during hospitalization.
6. **Treatment details**: Information on the medications prescribed, including type, dosage, and duration.
7. **Outcome data**: Details of the patient’s recovery, mainly in-hospital mortality.

### Data preparation

Before starting ML modeling, we cleaned the medical files records that we collected. At the beginning we had 587 patient files with pneumonia diagnosis. After we removed the files with HAP, ventilator-associated pneumonia (VAP) and duplicated CAP files we reduced the number to 412 cases.

The files at the beginning had multiple lab results and vital signs records taken over the LOS in the hospital of the patients. In our study the aim is to make a rapid decision about the clinical situation of the patients within few hours of the admission, so we had got only the first records for the lab results and vital signs reading (the number of features reduced from 3562 to 665 features).

We conducted data preprocessing steps over the left CAP files after cleaning, involved handling missing values- some files had high percentage of missing data, so we dropped them and finally reached 251 patients files. Other files with low percentage of missing, we normalized continuous variables and encoding categorical variables. Libraries such as Pandas and NumPy were employed to make data manipulation more efficient, ensuring consistency and accuracy. The normal ranges were used to handle missing values.

To optimize the dataset, columns with a single value or where 95% or more of the entries were identical were removed. These columns provided little to no meaningful information when analyzing correlations with the target variable. Their inclusion would have unnecessarily increased the dimensionality of the data, introducing low variance, adding noise, and potentially leading to biased results or overfitting. By eliminating these columns, the dataset was simplified, reducing noise and improving its overall quality for subsequent analysis. The number of features now is 132 features. This step is called features selection.

### ML techniques and feature evaluation

ML techniques were applied to analyze the collected data and develop predictive models for CAP’s etiology and mortality outcomes. The selection of appropriate ML algorithms is crucial for handling the complexity and variety of the data involved. These techniques were chosen based on their proven effectiveness in similar healthcare datasets [22]. This section outlines the methodology used to evaluate the impact of the most important features on the target variable using seven distinct approaches: linear regression, random forest, mutual information analysis, Lasso regression, logistic regression, Shapley additive explanations (SHAP) values, and correlation analysis. Each method provides unique insights into the relationship between the features and the target variable. The dataset was randomly divided into training and testing subsets using an 80:20 split ratio. Additionally, model performance and generalizability were assessed using 5-fold cross-validation within the training data. The average performance metrics across the folds were reported to ensure robustness.

#### Linear regression coefficients

Linear regression was employed to understand the linear relationships between each feature and the target variable. The coefficients derived from the model indicate the direction and magnitude of the feature’s impact [23, 24]. This analysis provides a straightforward interpretation of feature contributions in a linear framework.

#### Random forest

A random forest model was used to assess the relative importance of features in predicting the target variable. Feature importance scores were computed, indicating how critical each feature is for model predictions [25–27]. Features with higher importance scores play a more significant role in predictions, while those with lower scores have a minor impact. Unlike linear regression, which assumes linear relationships between variables, Random Forest can capture complex, non-linear relationships, providing a complementary perspective on feature importance.

#### Mutual information analysis

Mutual information analysis is a statistical technique used to measure the dependence between two variables. It quantifies how much information knowing one variable provides about the other [28]. Rooted in information theory, mutual information is widely used in fields like ML, data analysis, and bioinformatics. In the medical field, mutual information serves as a valuable tool for analyzing the relationship between medical predictors (such as biomarkers, test results, and demographic data) and health outcomes (such as disease presence, survival rates, or treatment effectiveness). By quantifying the dependency between predictors and outcomes, mutual information aids in tasks like feature selection, diagnostic modeling, and risk stratification [29].

#### Least absolute shrinkage and selection operator (Lasso) regression

Lasso is a type of linear regression that performs both feature selection and regularization to enhance prediction accuracy and interpretability. It is a statistical technique that can be used to study the effects of clinical variables in outcome prediction [30].

#### Logistic regression

Logistic regression is a widely used statistical and ML technique in clinical research for predicting binary outcomes, such as disease presence (yes/no), treatment success (effective/ineffective), or survival (alive/deceased). It estimates the probability that a given input belongs to a particular class based on clinical predictors (e.g., age, blood pressure, and cholesterol levels).

#### SHAP values

SHAP values were used to explain the contribution of individual features to the model’s predictions. SHAP analysis was employed not as a standalone predictive model but as a post hoc interpretability tool to explain the output of the trained random forest model. The SHAP values quantified the contribution of individual features to the model’s predictions, enhancing the interpretability and transparency of the decision-making process. This method provides detailed insights into both the magnitude and direction of feature impact: blue points represent lower feature values that contribute less to the target, while red points represent higher feature values that contribute more. An SHAP summary plot was generated to visualize the overall influence of each feature on the target variable. This method is particularly useful for verifying the impact direction indicated by linear models and for uncovering interactions and non-linear relationships [31].

#### Correlation with the target

Correlation analysis was conducted to measure the linear relationship between each feature and the target variable [24]. Positive correlation indicates that as the feature increases, the target variable also increases. Whereas, negative correlation indicates that as the feature increases, the target variable decreases.

While correlation provides a simple measure of association, it does not account for non-linear relationships or interactions between features [24].

Each model was trained and validated on separate data splits to assess their performance accurately. Cross-validation techniques ensure that the models generalize well on unseen data. The performance of each method was measured using appropriate metrics such as accuracy, sensitivity, specificity, and area under the receiver operating characteristic (ROC) curve.

**Figure 1.**
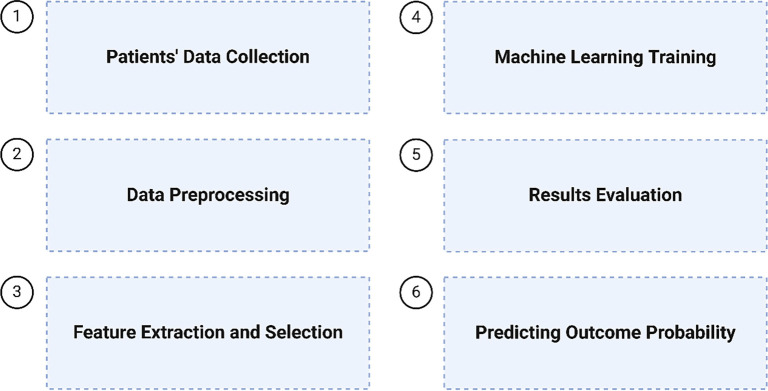
**Overview of the six-step machine learning workflow for predicting outcome probabilities**.

To have an accurate and unbiased model, we made sure that our dataset is balanced. A balanced dataset with an equal number of observations for both recovered and dead patients was created to train and test our model. The data samples (patients) in the training dataset have been selected randomly and they were completely separated from the testing data (Figure 1).

Prior to balancing, the dataset exhibited a class imbalance with a higher proportion of survivors compared to non-survivors. To solve this issue, a random oversampling of the minority group (survivors) was performed to achieve a 1:1 class distribution in the training set. This resampling was conducted exclusively on the training data to avoid information leakage and ensure a fair evaluation of the model’s performance.

### Tools and software

Analysis was conducted using Python programming language in a Jupyter Notebook environment. Key libraries utilized include:

Pandas: For data manipulation and cleaning.

NumPy: For numerical operations.

Matplotlib/Seaborn: For data visualization.

These tools ensured the efficient handling of large datasets and the generation of high-quality visualizations to identify patterns and outliers.

### Data transformation

After removing unnecessary columns, the dataset was further refined by encoding categorical variables. Columns with string values were identified, and a LabelEncoder was applied to convert them into numeric values. With the number of columns reduced from 665 to 132, LabelEncoder was chosen for its simplicity and efficiency, especially for columns with only two categories. This approach helped maintain the dataset’s simplicity, avoiding additional complexity that could have posed challenges in analysis and modeling.

### Exploratory data analysis (EDA)

EDA was performed to understand the distribution of variables, detect anomalies, and assess correlations between features. Statistical summaries were computed to identify key trends, and graphical methods such as bar charts, heatmaps and SHAP Summary Plots were generated.

### Feature selection

A total of 132 features were selected from the original dataset in a process called feature selection. All relevant features were extracted during this step. The primary goal of feature selection is to identify the most informative features while removing redundant ones, thereby reducing the model’s dimensionality and complexity. The selected features included demographic data, clinical characteristics, and laboratory results collected at the time of admission. Predictor variables consisted of age, gender, vital signs (e.g., blood pressure, heart rate, respiratory rate, and temperature), the number of medical comorbidities (such as hypertension and diabetes mellitus [DM]), laboratory data (including Complete Blood Count, Comprehensive Metabolic Panel, and arterial blood gas (ABG) tests), and clinical scores (such as pneumonia severity index (PSI) and CURB-65). Table 1 presents these features.

**Table 1:** Features utilized in the machine learning algorithm

Demographic	Age
	Gender		
Clinical finding	Number of hospitalizations	Number of pneumonia	Temperature
	Pulse	Respiratory rate	SBP
	DBP	LOS	PSI class
	Oxygen saturation	Oxygen therapy	PSI
	CURB-65	Initial finding fever	Initial finding shortness of breath
	Location	ICU length of stay	
Laboratory results	Glucose serum	Hemoglobin A1C (HbA1c)	Random blood glucose
	PaO_2_	PCO_2_	HCO_3_
	ABG BE	Albumin serum	CPK
	BUN	Alkaline phosphatase	ALT
	AST	Total Bilirubin	Direct Bilirubin
	Amylase serum	pH	Hemoglobin
	HCT	MCH	MCV
	MCHC	PDW	Platelet count
	WBC	RDW	MPV
	RBC	ESR	Lymphocytes count
	Monocytes count	Eosinophils count	Basophils count
	Neutrophils count	Magnesium serum	Potassium serum
	Calcium serum	Sodium serum	Total protein
	SGOT	SGPT	Creatinine serum
	Urine PH	CRP	PCT
	Uric acid	HDL cholesterol	LDL cholesterol
	Prothrombin time	D-dimer	INR
	LDH	Troponin	aPTT
	Culture	Sputum culture	Urine character hazy
	PCR		
Medical history	DM	Hypertension	CVD
	Alzheimer	COVID-19	Hypotension
	Septic shock	Cough	Dry cough
	Chest pain		
Medication	Imipenem	Cefepime	Vancomycin
	Meropenem	Ceftriaxone	Levofloxacin
	Susceptibility Imipenem	Susceptibility Piperacillin tazobactam	Imipenem cilastatin
	Susceptibility Meropenem	Susceptibility Cefepime	Susceptibility Levofloxacin
	Susceptibility Ceftazidime	Susceptibility Aztreonam	Susceptibility Ciprofloxacin
	Susceptibility Amikacin	Amlodipine	Aspirin
	Bisoprolol	Vitamin C	Atorvastatin
	Cilastatin	Furosemide	Lansoprazole
	Enoxaparin	Insulin basal	Lactulose
	Enalapril	Insulin bolus	Omeprazole
	Prednisolone	Tocilizumab	Anti-platelet
	Zinc	Antibiotics	Anti-coagulant
	B complex	Antiviral	Number of PRN medications
	mAb tocilizumab	Number of medications	
	Number of regular medications	Guideline concordant antibiotics	

Abbreviations: SBP: Systolic blood pressure; DBP: Diastolic blood pressure; LOS: Length of stay; PSI: Pneumonia severity index; CURB-65: Confusion, urea, respiratory rate, blood pressure, age ≥65; ICU: Intensive care unit; HbA1c: Hemoglobin A1C; PaO_2_: Partial pressure of oxygen; PaCO_2_: Partial pressure of carbon dioxide; HCO_3_: Bicarbonate; ABG BE: Base excess of arterial blood gas; CPK: Creatine phosphokinase; BUN: Blood urea nitrogen; ALT: Alanine aminotransferase; AST: Aspartate aminotransferase; SGOT: Serum glutamic-oxaloacetic transaminase; SGPT: Serum glutamic-pyruvic transaminase; MCV: Mean corpuscular volume; HCT: Hematocrit; MCH: Mean corpuscular hemoglobin; MCHC: Mean corpuscular hemoglobin concentration; PDW: Platelet distribution width; WBC: White blood cell count; RDW: Red blood cell distribution width; MPV: Mean platelet volume; RBC: Red blood cell count; ESR: Erythrocyte sedimentation rate; PCR: Polymerase chain reaction; PCT: Procalcitonin; CRP: C reactive protein; HDL: High-density lipoprotein; LDL: Low-density lipoprotein; INR: International normalized ratio; LDH: Lactate dehydrogenase; aPTT: Activated partial thromboplastin time; DM: Diabetes mellitus; CVD: Cardiovascular disease; PRN: Per registered nurse (as needed).

### Statistical analysis

Statistical analyses were performed, followed by the development of predictive models to forecast key variables.

The statistical analysis starts with examining the data collected through descriptive statistics to identify the central tendencies, normality distribution (using Shapiro–Wilk test, *Q*–*Q*, and distribution plots), and variability. Continuous variables were summarized using means and standard deviations (SD) or medians and interquartile ranges (IQR), depending on normality assessment, in addition to frequencies and percentages for categorical variables. The statistical analysis was conducted using JASP 18.3.0 (Jeffreys’s Amazing Statistics Program) and IBM SPSS statistics 25.

## Results

### Demographic and clinical characteristics

A sample of 251 participants was included in this study. The median for their age was 60 years (IQR ═ 25). Most were male 146 (58.2%) and 105 (41.8%) were female. The majority of patients were admitted to the ICU (221, 88.0%) compared to the Ward (30, 12.0%). The median LOS for all patients was 10 days (IQR 13). For patients in the ICU, the median ICU LOS (ICU_LOS) was 4.77 days (IQR 1.77).

Regarding COVID-19 status, 18 (7.2%) had a current SARS-CoV2 infection, and 9 (3.6%) had a previous history of COVID-19 (Table 2).

**Table 2:** Demographic and clinical data of participants (*n* ═ 251)

**Feature**	**Frequency**	**Valid percentage %**	**Median (IQR)**
Gender			
Age (years)			60 (IQR = 25)
Location			
Ward	30	12.0	
ICU	221	88.0	
LOS			10 days (IQR = 13)
ICU_LOS			4.77 days (IQR = 1.77)
Current COVID_19	18	7.2	
Previous COVID_19	9	3.6	
Diseases			
DM	76	30.3	
Septic shock	64	25.5	
HTN	33	13.1	
CVD	22	8.8	
Alzheimer’s disease	17	6.8	
Another lung disease	14	5.6	
CKD	12	4.8	
Anemia	7	2.8	
Asthma	6	2.4	
COPD	5	2.0	
Hypotension	5	2.0	
GERD	5	2.0	
Dyslipidemia	4	1.6	
History			
Number of previous pneumonias			0.77 (IQR = 0)
Number of previous hospitalizations			2.4 (IQR = 3.7)
Kidney transplantation	4	1.6	

Abbreviations: ICU: Intensive care unit; LOS: Length of stay; ICU_LOS: Intensive care unit length of stay; DM: Diabetes mellitus; HTN: Hypertension; CVD: Cardiovascular disease; CKD: Chronic kidney disease; COPD: Chronic obstructive pulmonary disease; GERD: Gastroesophageal reflux disease.

Participants had a high prevalence of comorbidities, with DM being the most prevalent (30.3%), followed by septic shock (25.5%), hypertension (13.1%), and cardiac diseases (8.8%). Other reported conditions were Alzheimer’s disease (6.8%), chronic kidney disease (4.8%), anemia (2.8%), asthma (2.4%), and chronic obstructive pulmonary disease (COPD) (2.0%) (Table 2).

### Treatment background characteristics

The chronic treatments varied, with vitamin C and zinc supplements showing the highest rates of use, due to COVID-19 recommendations at the time for their prophylactic and supportive effects (29.1% and 27.5%, respectively). Insulin therapy was also used, with 24.7% of patients receiving insulin bolus and 15.9% on basal insulin, alongside DM as the most common chronic condition. Other frequently used medications included omeprazole (19.1%), bisoprolol (17.9%), and B complex vitamins (16.3%) (Table 3). The most common short-term treatments were antibiotics (80.1%), anticoagulants (78.1%), and enoxaparin (77.7%). Antiviral agents were used by a small proportion of patients (8.0%), and only 4.8% received supportive drugs, including albumin, favipiravir, and norepinephrine injections (Table 3).

**Table 3:** Descriptive data related to treatment

**Feature**	**Frequency**	**Valid percentage**
*Chronic treatment*		
Vitamin C	73	29.1
Zinc	69	27.5
Insulin bolus	62	24.7
Omeprazole	48	19.1
Bisoprolol	45	17.9
B complex	41	16.3
Insulin basal	40	15.9
Antiplatelet	39	15.6
Aspirin	38	15.1
Amlodipine	31	12.4
Furosemide	31	12.4
Atorvastatin	27	10.8
Lactulose	24	9.6
Lansoprazole	21	8.4
Enalapril	17	6.8
Prednisolone	16	6.4
MAb_tocilizumab	16	6.4
Tocilizumab	16	6.4
Ipratropium inhaler	13	5.2
Clopidogrel	12	4.8
Hydrochlorothiazide	12	4.8
Azithromycin	11	4.4
Piperacillin - Tazobactam	11	4.4
Budesonide inhaler	10	4.0
Vitamin D	10	4.0
Metformin	9	3.6
Colchicine	9	3.6
Ca carbonate	7	2.8
Candesartan	7	2.8
Metronidazole	7	2.8
Valsartan	7	2.8
Bromazepam	6	2.4
Carvedilol	4	1.6
*Short-term treatment*		
Antibiotics	201	80.1
Anticoagulant	196	78.1
Enoxaparin	195	77.7
Antiviral agent	20	8.0
Albumin	12	4.8
Favipiravir	12	4.8
Norepinephrine injection	12	4.8
Paracetamol	11	4.4
Guaifenesin	11	4.4
Dextrose injection	11	4.4
Morphine	8	3.2
Remdesivir injection	8	3.2

**Table 4:** Descriptive data on pneumonia

**Feature**	**Frequency**	**Valid percentage**
*PSI class*		
1	48	19.1
2	102	40.6
3	58	23.1
4	33	13.2
5	10	4.0
*CURB 65*		
0	47	18.7
1	132	41.8
2	62	24.7
3	10	4.0
*Bacterial infection*		
Yes	215	85.7
No	36	14.3
*Outcome*		
Survivor	169	67.3
Non survivor	82	32.7
*Initial findings*		
SOB	97	38.6
Fever	41	16.3
Productive cough	17	6.8
Dry cough	16	6.4
Pleuritic chest pain	16	6.4
Headache	11	4.4
Vomiting	11	4.4
Abdominal pain	10	4.0
Intubation/mechanical ventilation	10	4.0
Diarrhea	10	4.0
Fatigue	8	3.2
Nausea	6	2.4
Constipation	5	2.0
Muscular or joint pain	4	1.6
Loss of taste and smell	4	1.6

Abbreviations: PSI: Pneumonia severity index; CURB 65: Confusion, urea, respiratory rate, blood pressure, Age ≥65; SOB: Shortness of breath.

**Table 5:** Performance metrics of logistic regression models in predicting mortality and bacterial infections

**Metric**	**Mortality outcome (training set)**	**Mortality outcome (test set)**	**Bacterial infection outcome (training set)**	**Bacterial infection outcome (test set)**
Accuracy	0.88	0.86	0.98	0.95
AUROC	0.90 (95% CI: 0.85–0.95)	0.88 (95% CI: 0.83–0.93)	0.99 (95% CI: 0.97–1.00)	0.96 (95% CI: 0.92–0.98)
Sensitivity	0.95	0.94	1	1
Specificity	0.85	0.8	0.95	0.9
PPV	0.9	0.88	0.97	0.95
NPV	0.92	0.91	0.98	0.96

Abbreviations: AUROC: Area under the receiver operating characteristic curve; PPV: Positive predictive value; NPV: Negative predictive value.

**Table 6:** Comparative variable importance

**Feature**	**Mutual information**	**LASSO coefficient**	**Logistic regression odds ratio**	**Mean SHAP value**
Zinc	✓	–	–	✓
Vitamin C	✓	–	–	✓
Enoxaparin	✓	–	✓	✓
Neutrophils	✓	✓	–	✓
Meropenem	–	✓	–	✓
ICU Stay	–	–	✓	✓
pH	–	✓	✓	✓
WBC	✓	✓	✓	✓
Platelets	✓	✓	✓	✓

Abbreviations: ICU: Intensive care unit; WBC: White blood cell count.

### Characteristics specific to pneumonia

Among the included participants, the majority were classified as Class 2 (40.6%) and Class 3 (23.1%) according to the PSI, indicating moderate severity. Based on CURB-65 scores, 41.8% of patients had a score of 1, and 24.7% had a CURB-65 score of 2, indicating differences in pneumonia risk levels.

The majority of patients got bacterial CAP with 215 (85.7%), most of patients were survived 169 patients (67.3%) (Table 4).

The most common initial clinical symptoms were dyspnea (38.6%) and fever (16.3%). Other symptoms were productive cough (6.8%), dry cough (6.4%), pleuritic chest pain (6.4%), headache (4.4%), vomiting (4.4%), and abdominal pain (4.0%). Notably, 4.0% of patients required intubation or mechanical ventilation (Table 4).

### ML analysis results to predict mortality

#### Global feature correlation structure

A comprehensive correlation heatmap visualized the interdependencies across all features. This analysis revealed a dense correlation structure, indicating significant overlap among variables. The redundancy identified highlights the need for preprocessing steps, such as feature selection, to ensure model robustness and reduce overfitting. This step is performed after cleaning the data by excluding columns with all null values, leading to a more accurate and reliable correlation matrix. The diagonal of the heatmap typically contains values of 1, representing the perfect correlation of each feature with itself. The color intensity in each cell reflects the strength and direction of the correlation between the corresponding features on the axes. A color gradient from blue to red is used: positive correlations (closer to 1) are shown in warm colors (e.g., red), while negative correlations (closer to −1) appear in cool colors (e.g., blue). Certain feature groups exhibit noticeable correlations, suggesting potential multicollinearity or shared variance. For example, tightly clustered red squares indicate high correlations between related clinical markers or treatment variables, while patches of blue represent negative associations between certain features. This matrix emphasizes the importance of addressing multicollinearity during model development and suggests that specific feature groups may have overlapping predictive power. Identifying and managing these relationships can ultimately enhance model performance and interpretability.

#### Feature correlation analysis

**Figure 2.**
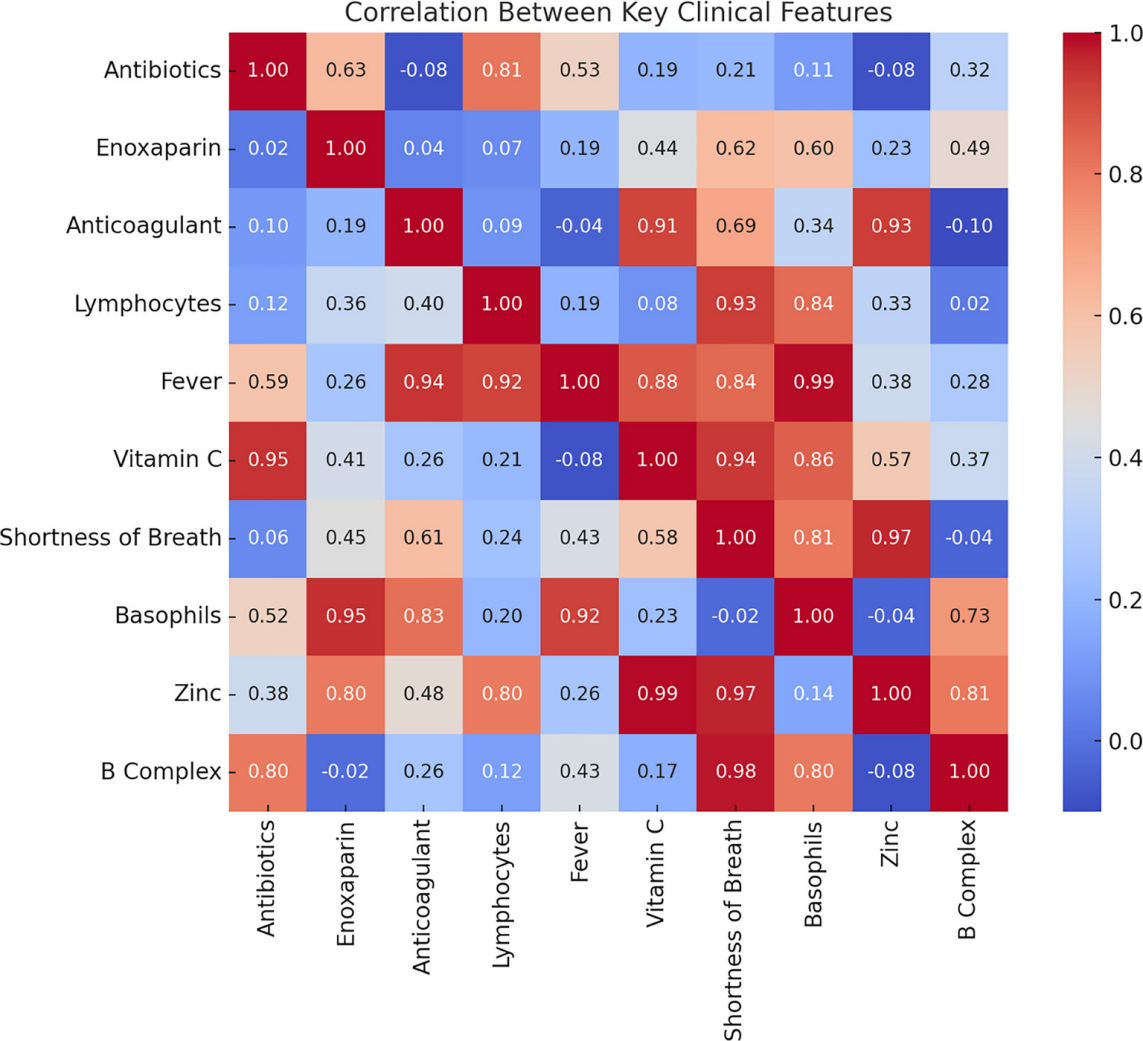
**Heatmap of strongly correlated features.** Pearson correlation coefficients: A heatmap of strongly correlated features. Warm colors represent higher correlations, while cool colors indicate negative correlations. Strong positive correlations are observed between inflammatory markers (e.g., C-reactive protein [CRP], ferritin) and between white blood cell (WBC) count and neutrophil count (*r* > 0.7). This pattern suggests potential collinearity among markers of systemic inflammation, which may affect model stability and motivate variable selection or regularization strategies.

A correlation matrix is often calculated before building predictive models in Python (or any other programming language) to gain insights into the relationships between features. Highly correlated features can cause multicollinearity, which may require dimensionality reduction techniques like principal component analysis (PCA) or the elimination of redundant features to enhance model performance and interpretability. In our study, the correlation matrix was particularly useful for identifying relationships between different features in the dataset. It shows how strongly each feature is related to others, which is crucial for understanding the underlying structure of the data. We used the correlation matrix to identify pairs of features with a correlation score of 0.8 or higher (i.e., highly correlated features). These pairs were then saved into a CSV file for easier inspection. To evaluate the internal structure of the dataset and identify potential predictors of mortality, we generated Pearson correlation heatmaps. In our analysis, a high coefficient indicates a strong association with the survival rate. During the encoding process for categorical variables, we assigned a value of 1 for the survival outcome and 2 for the non-survival outcome. Thus, the correlation reflects how closely each feature is associated with the survival outcome, with higher values indicating a stronger relationship. This correlation pertains specifically to the survival rate in this context. Figure 2 shows the intercorrelation matrix among all features, revealing strong positive correlations between inflammatory markers (e.g., C-reactive protein [CRP], ferritin) and between WBC count and neutrophil count (*r* > 0.7). These findings suggest potential collinearity among markers of systemic inflammation, which could affect model stability. This may necessitate variable selection or regularization strategies.

#### Top correlated features with the mortality target

**Figure 3.**
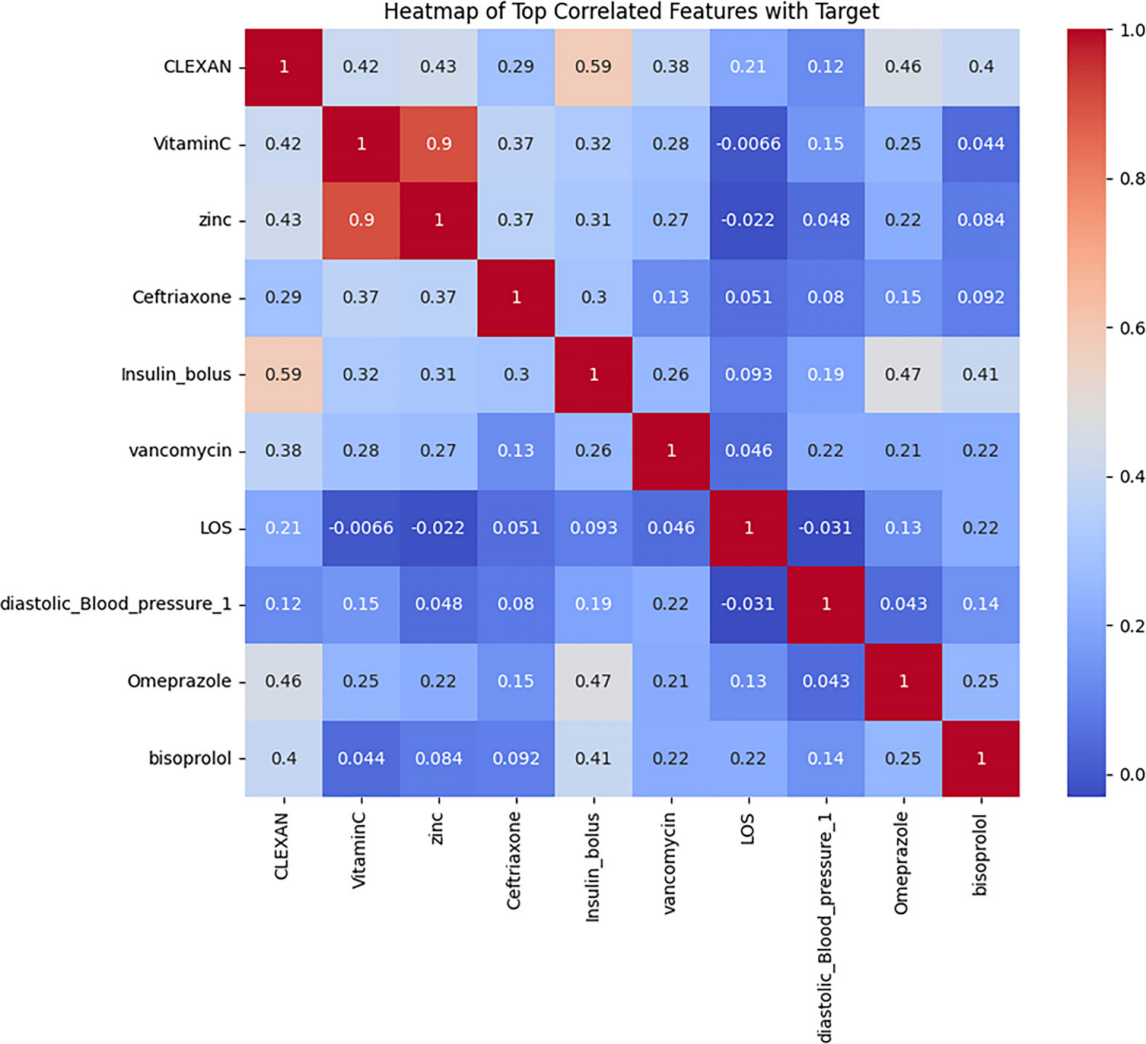
**Heatmap of top correlated features with mortality outcomes.** Pearson correlation coefficients: Heatmap of features and target outcome. Warm colors represent higher correlations, while cool colors indicate negative correlations. Top positive correlates: age (*r* ≈ 0.45), neutrophils (*r* ≈ 0.41), CRP (*r* ≈ 0.39), ferritin (*r* ≈ 0.36); top negative correlates: lymphocytes (*r* ≈ −0.42), oxygen saturation (*r* ≈ −0.38), hemoglobin (*r* ≈ −0.33). A focused panel also highlights high correlations for vitamin C, zinc, enoxaparin (CLEXAN), and insulin bolus. Abbreviation: LOS: Length of stay.

Figure 3 shows the correlation between individual features and the target outcome of mortality. Age (*r* ≈ 0.45), neutrophil count (*r* ≈ 0.41), CRP (*r* ≈ 0.39), and ferritin (*r* ≈ 0.36) were among the strongest positive correlates with mortality, indicating their potential as high-risk predictors. Conversely, lymphocyte count (*r* ≈ −0.42), oxygen saturation (*r* ≈ −0.38), and hemoglobin (HGB) level (*r* ≈ −0.33) showed the strongest negative correlations, suggesting a protective role. These patterns underscore the prognostic relevance of age and markers of inflammation and hypoxia in predicting adverse outcomes. The identification of these features supports their prioritization in model development and interpretability analysis. A focused heatmap (Figure 3) showed the variables most correlated with the mortality Notably, vitamin C, zinc, enoxaparin (CLEXAN) and insulin bolus exhibited the highest correlations.

Zinc and Vitamin C exhibited the highest positive correlation (*r* ═ 0.90), suggesting a strong relationship between these two variables with survival rate. Whereas, Insulin Bolus and Enoxaparin demonstrated a moderate correlation (*r* ═ 0.59), highlighting a potential interaction. LOS showed minimal correlation with most variables, with the highest being a weak negative correlation with Vitamin C (*r* ═ –0.0066). Diastolic blood pressure shows low correlations with other variables, with the maximum being a weak positive correlation with Vitamin C (*r* ═ 0.15).

#### Mutual information analysis

**Figure 4.**
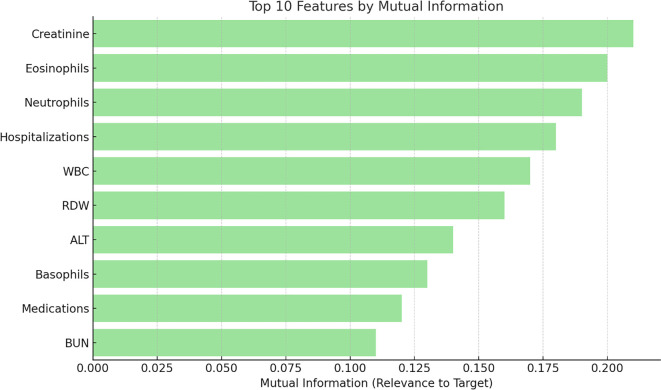
**Mutual information of features related to mortality outcomes.** Mutual information scores quantify each feature's dependency on mortality, producing a ranked list of informative predictors. Top contributors include creatinine, WBC (including eosinophils), and neutrophil count; the number of previous hospitalizations also ranks highly. RDW and ALT are additional significant features. Overall, inflammatory markers—eosinophils, neutrophils, and basophils—show high informativeness, underscoring their value for model prioritization and clinical decision-making. Abbreviations: WBC: White blood cell count; RDW: Red blood celldistribution width; ALT: Alanine aminotransferase.

The mutual information scores (Figure 4) quantify the dependency between each feature and the target variable (mortality), providing a ranking of the most informative predictors. Key findings include creatinine concentration, WBC including eosinophils, and neutrophils count. Number of previous hospitalizations is also a top contributor. Red-cell distribution width (RDW) and alanine aminotransferase (ALT) are other significant features reflecting their clinical relevance. This analysis underscores the critical role of inflammatory markers (eosinophils, neutrophils, and basophils count) in mortality outcome prediction, highlighting their importance in clinical decision-making.

#### Feature coefficients from Lasso regression

**Figure 5.**
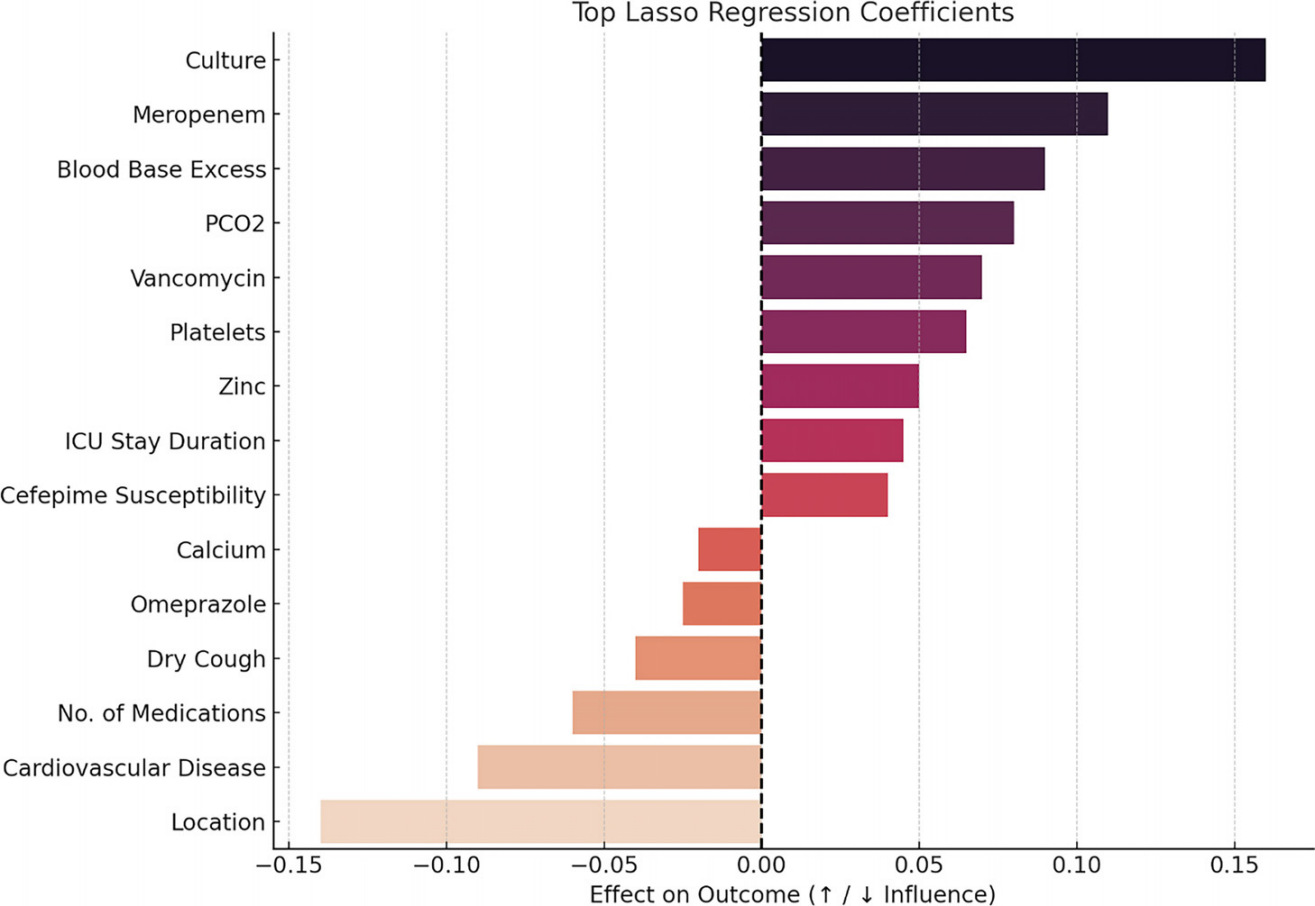
**Top feature coefficients from Lasso regression.** Coefficients indicate each variable's direction and magnitude of association with mortality. “Culture” shows the largest positive coefficient (β ≈ 0.15) but is not clinically meaningful (test-performed indicator). Meropenem has a strong positive coefficient; ABG variables (pH, Base Excess, PCO_2_) and platelet count also contribute positively, underscoring their relevance for risk prediction. Abbreviation: ICU: Intensive care unit.

The Lasso regression analysis (Figure 5) refined the list of predictors by assessing the magnitude and direction of their contributions to mortality risk. Among the variables, “culture” showed the highest positive coefficient (0.15). However, the culture variable is not clinically significant, as it merely indicates whether the patient underwent a culture test or not. Meropenem demonstrated a significant positive coefficient, highlighting its importance in predicting mortality. Additionally, ABGs (Base Excess, PCO_2_) and platelet count all contributed positively to the model. This analysis emphasizes the critical roles of meropenem, ABGs, pH, and PCO_2_ in predicting mortality outcomes.

#### Feature importance from logistic regression

**Figure 6.**
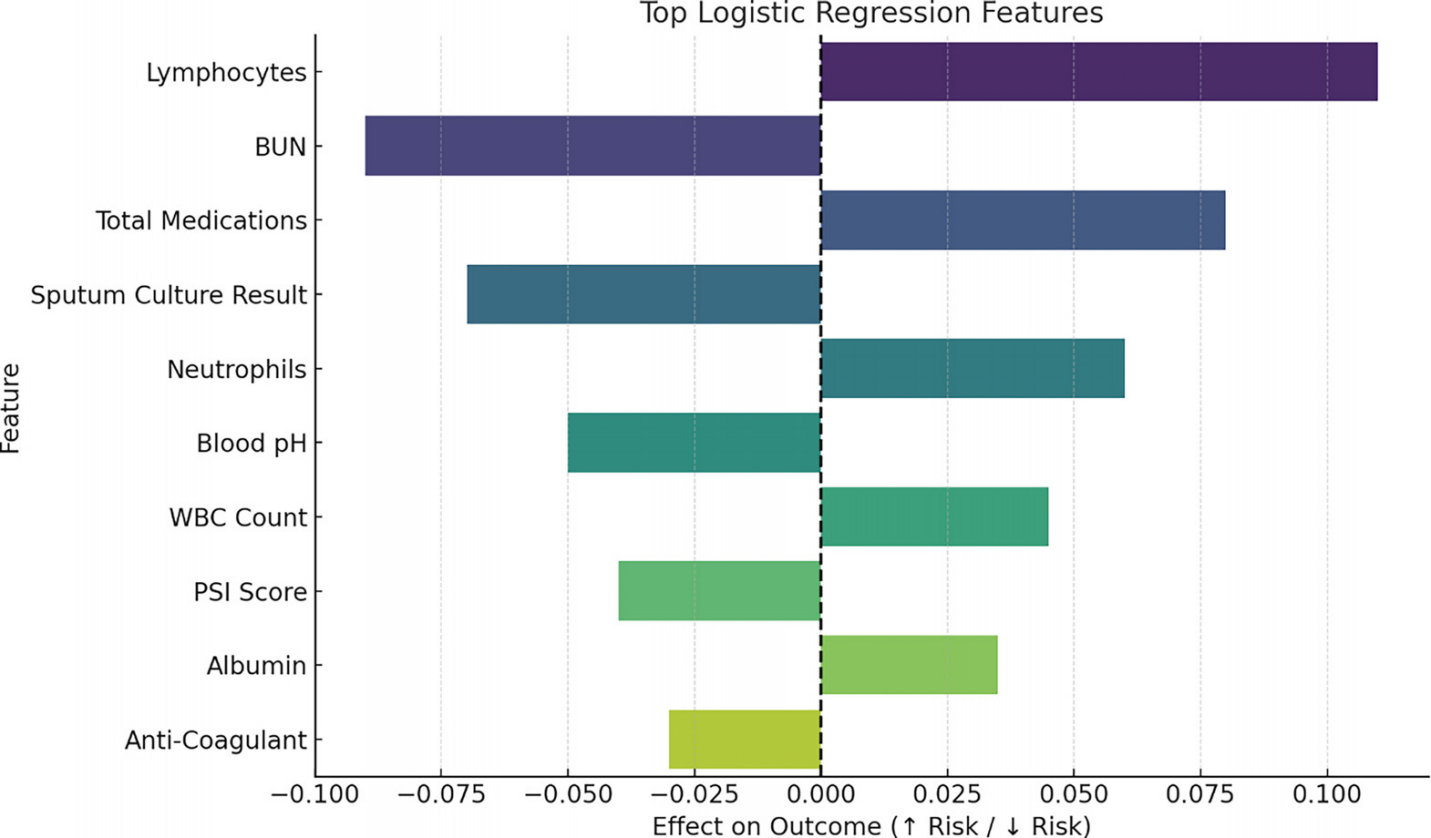
**Feature importance from logistic regression.** Coefficients indicate direction and strength. pH is the most impactful predictor with a strong negative coefficient (≈ −1.0). ICU_LOS and LOS show substantial positive effects (≈ 0.6 and ≈ 0.4), indicating longer stays are linked to higher mortality. Additional important contributors include PCO_2_, albumin, WBC, bicarbonate (HCO_3_), and ABG measures. Abbreviations: BUN: Blood urea nitrogen; WBC: White blood cell count; PSI: Pneumonia severity index.

The logistic regression-based feature importance analysis (Figure 6) provided an additional perspective on variable significance. For instance, pH identified as the most impactful predictor with the strongest negative impact (coefficient approximately −1.0). ICU_LOS and LOS: Showed a substantial positive impact with a coefficient around 0.6, and 0.4 respectively, suggesting that longer stays are associated with higher mortality. This analysis underscores the critical roles for pH, LOS, ICU_LOS, PCO_2_, albumin, WBC, bicarbonate (HCO_3_) and ABGs in predicting mortality rate.

#### SHAP analysis of medication and laboratory influence on model predictions

**Figure 7.**
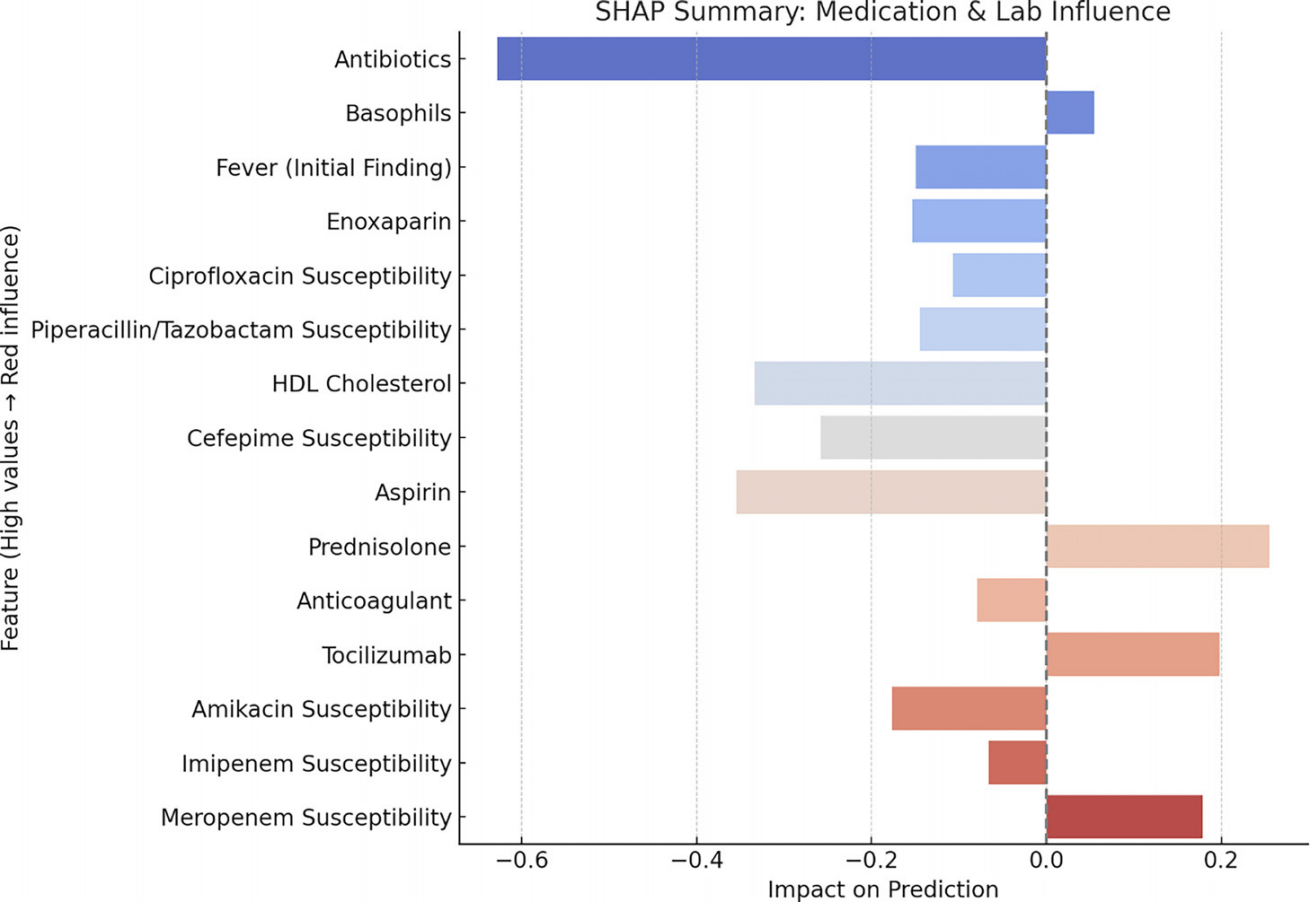
**SHAP summary plot: Analyzing the influence of medications and laboratory findings on model predictions.** Features are ranked by mean absolute SHAP values. Antibiotic use shows the strongest negative impact (SHAP < −0.6), with basophils and initial fever also lowering predicted risk. Enoxaparin and ciprofloxacin/piperacillin–tazobactam susceptibility align with lower risk, whereas meropenem/imipenem/amikacin susceptibility and tocilizumab, prednisolone, and anticoagulant use have positive impacts, likely reflecting greater disease severity. Abbreviations: HDL: High-density lipoprotein; SHAP: Shapley additive explanations.

The SHAP summary plot shown in Figure 7 illustrates the impact of individual features—primarily medications and laboratory findings—on the predictive model for survival outcomes. Features are ranked by their average absolute SHAP values, which represent their overall contribution to the model’s output. The most influential feature was antibiotic usage, which had a strong negative SHAP value (impact < –0.6), indicating that antibiotic administration significantly reduced the predicted risk of mortality. Basophil count and initial fever presentation also showed negative SHAP values, suggesting a modest inverse relationship with the predicted risk of mortality. Other features, such as enoxaparin, ciprofloxacin susceptibility, and piperacillin/tazobactam susceptibility, were associated with a lower predicted risk, possibly reflecting effective treatment or underlying microbial sensitivity. In contrast, meropenem susceptibility, imipenem susceptibility, and amikacin susceptibility had the largest positive impacts, potentially reflecting resistance to critical antibiotics or associations with more severe infections requiring these drugs. Among medications, tocilizumab, prednisolone, and anticoagulant use were positively associated with the outcome, suggesting these therapies may be markers of greater disease severity or higher baseline risk.

### ML results for predicting etiology of CAP

#### Correlation analysis for predicting bacterial infection

**Figure 8.**
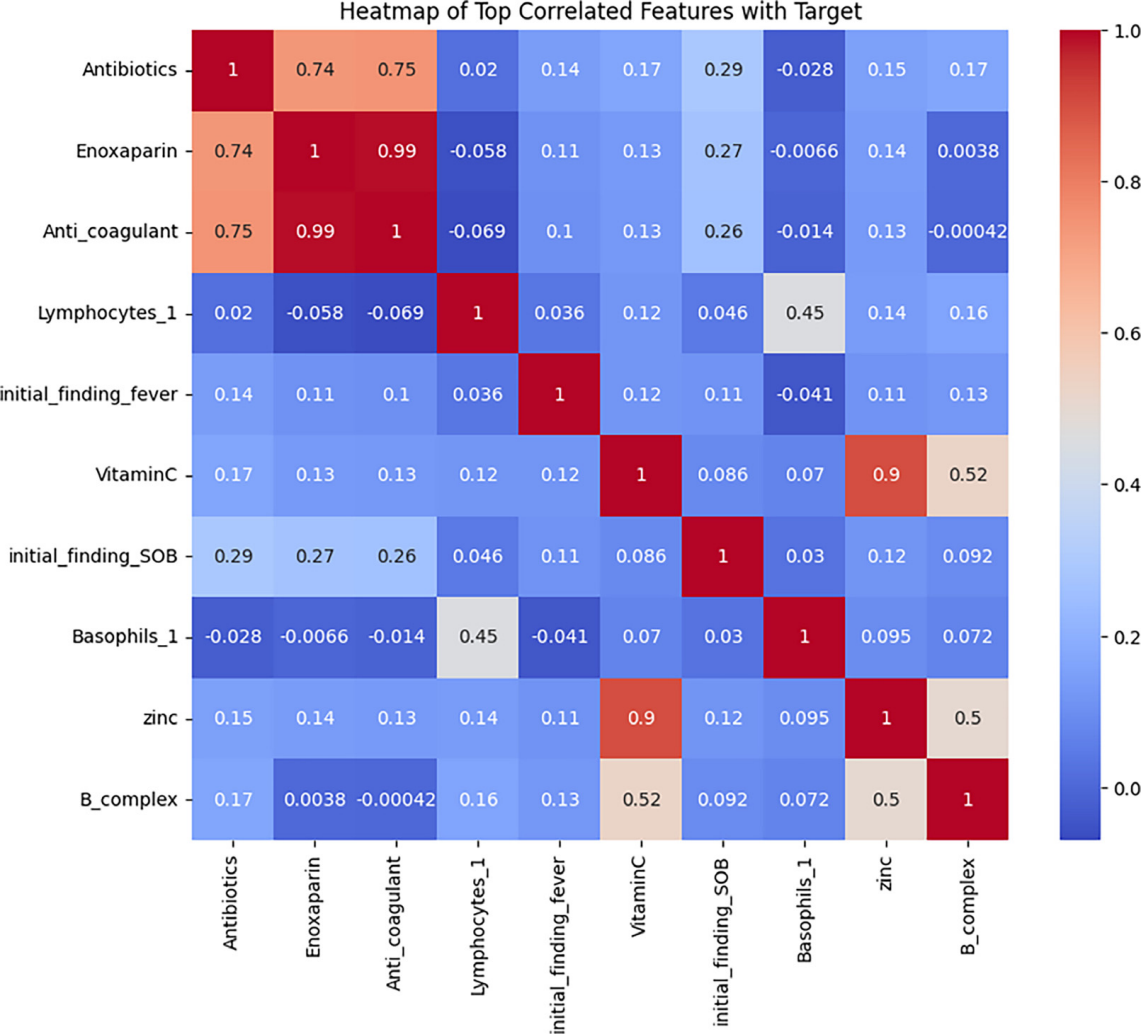
**Heatmap depicting correlations between clinical variables and bacterial infections.** Pearson correlation coefficients: Heatmap of features and target outcome. Warm colors represent higher correlations, while cool colors indicate negative correlations. The target was encoded as 1 for bacterial infection and 2 for no infection; thus higher coefficients indicate a greater likelihood of bacterial infection. This visualization supports ML-based etiology prediction in CAP. Abbreviations: SOB: Shortness of breath; CAP: Community-acquired pneumonia; ML: Machine learning.

A heatmap was generated to assess the correlation between clinical variables and bacterial infection (increasing coefficeints reading means higher opportunity for getting bacterial infection as the correlation was between the variables and positive bacterial infection result—Encoding bacterial infection with number one and no bacterial infection with number two) (Figure 8).

Enoxaparin, and other anti-coagulant treatments exhibited the strongest positive correlations with bacterial infection, with correlation coefficients of 0.99. Vitamin C supplementation showed also high correlation with Zinc (*r* ═ 0.90).

#### Feature importance analysis using logistic regression analysis for predicting bacterial infection

**Figure 9.**
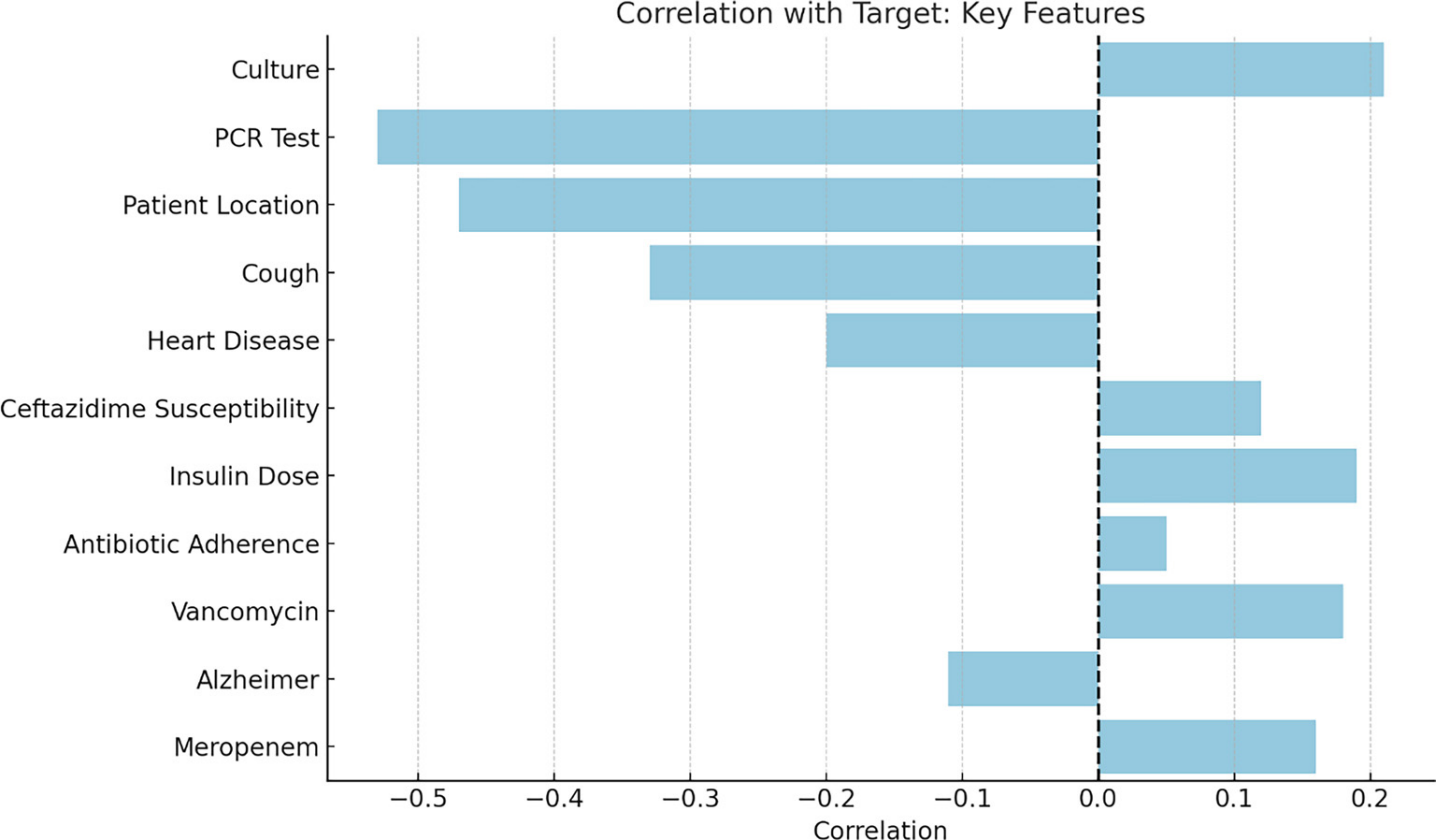
**Feature importance in logistic regression models.** The horizontal bar plot illustrates the coefficient of each feature, reflecting its actual contribution to the model’s predictions. A positive coefficient indicates a positive association with the target variable, while a negative coefficient signifies a negative association. Abbreviation: PCR: Polymerase chain reaction.

Logistic regression analysis was conducted to evaluate feature importance (Figure 9). Figure 9 presents the results of a logistic regression model showing the key features correlated with the bacterial infection. Vancomycin and Insulin Dose, are one of the most influential predictors, indicating that their presence was positively associated with the bacterial infection. Conversely, Cough, and Heart Disease had negative coefficients.

#### SHAP analysis

**Figure 10.**
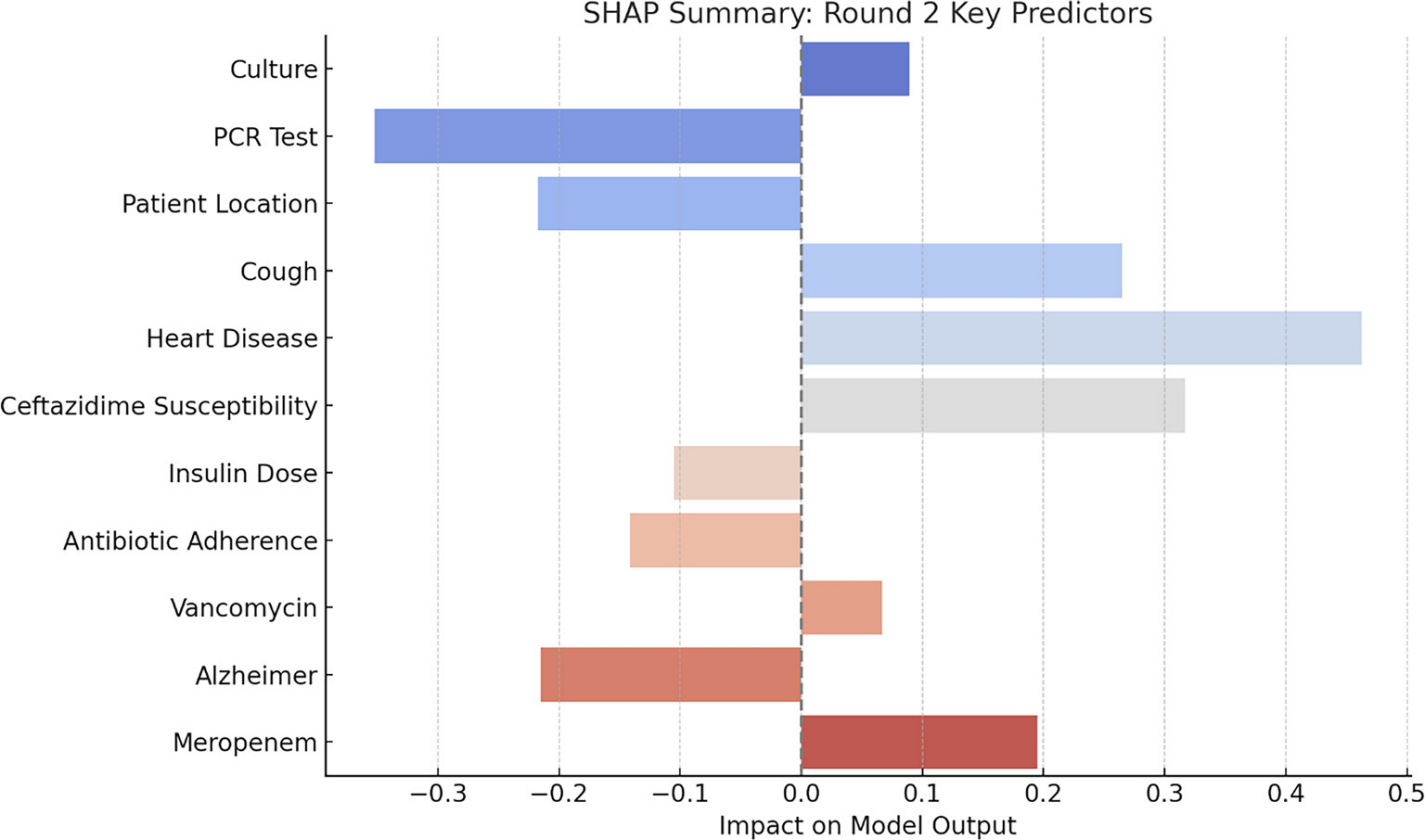
**SHAP summary plot: Analyzing feature impact on model output.** This figure presents the SHAP values that demonstrate the influence of various features on the model’s predictions. Positive SHAP values indicate a beneficial contribution to the outcome, whereas negative values reflect a detrimental impact. The color gradient, ranging from blue (indicating low feature values) to red (indicating high feature values), underscores the relationship between feature magnitude and model output. Notably influential features include Antibiotics, Basophils, and initial findings of fever, each exhibiting distinct effects based on their respective values. Abbreviations: SHAP: Shapley additive explanations; PCR: Polymerase chain reaction.

The SHAP summary plot (Figure 10) highlights the most influential predictors from the second round of model evaluation. Features such as Cough, Heart Disease, and Ceftazidime Susceptibility exhibited the highest positive SHAP values, indicating strong positive contributions to the model output. Conversely, PCR Test, Patient Location, and Culture had the most negative SHAP values, suggesting they were associated with a reduced predicted probability of the target outcome. Additionally, Meropenem, Alzheimer, Vancomycin, and Antibiotic Adherence showed moderate positive impacts, whereas Insulin Dose showed minimal negative influence. The color gradient further reveals that features with higher values (red) or lower values (blue) contribute differently to prediction magnitude, emphasizing the complex, value-dependent behavior of certain predictors in the model.

#### The correlation coefficients between selected features and the target variable

**Figure 11.**
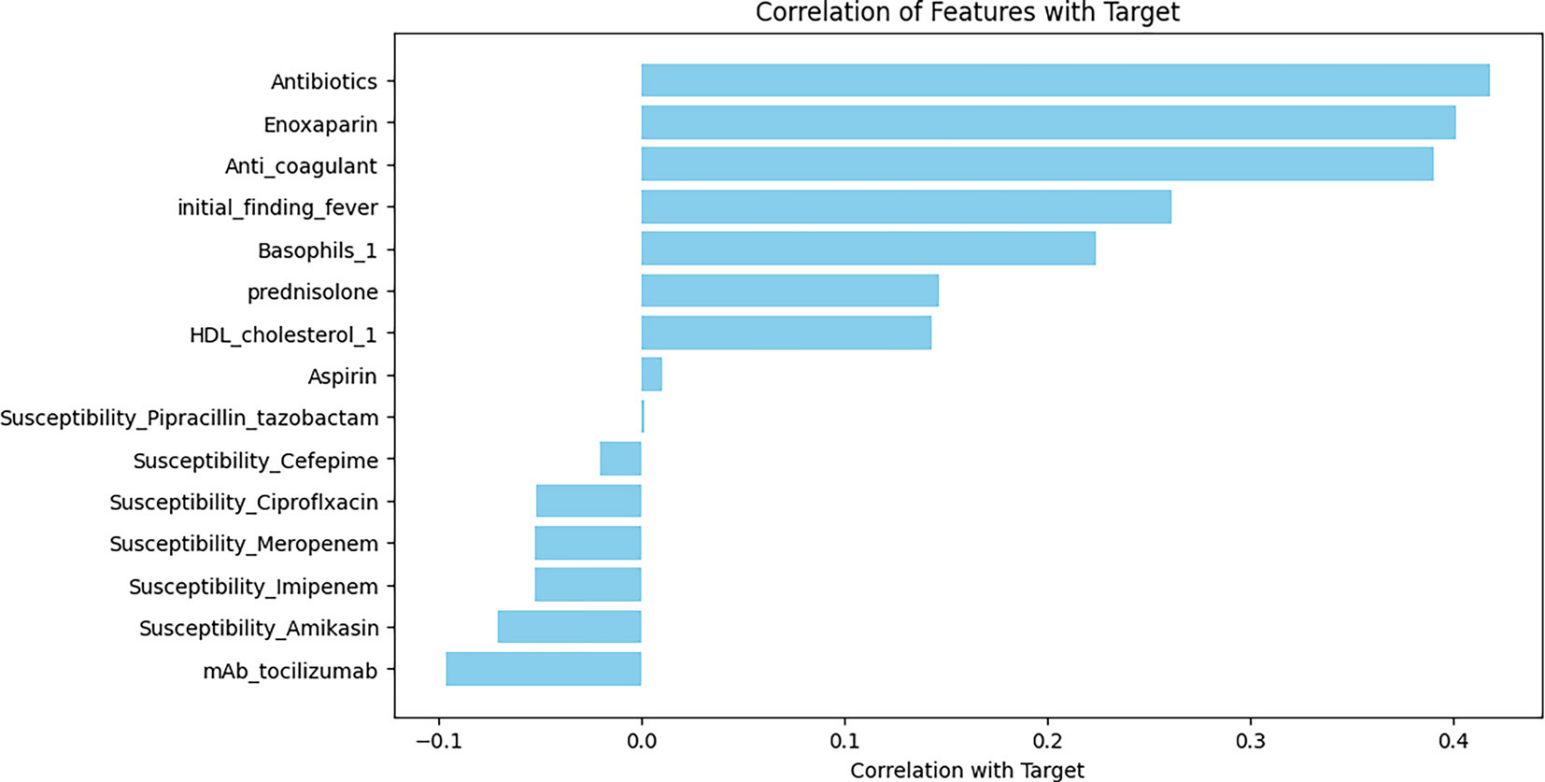
**Correlation of clinical and treatment features with bacterial infection as a primary outcome.** Antibiotics show the strongest positive correlation, with enoxaparin and anticoagulant use also positively associated; initial fever and basophils exhibit moderate positive correlations. In contrast, mAb tocilizumab and amikacin susceptibility show weak negative correlations, while other susceptibilities (e.g., cefepime, ciprofloxacin) are minimal or near zero. Abbreviation: HDL: High-density lipoprotein.

Figure 11 presents the correlation coefficients between selected features and the target variable. Antibiotics demonstrated the strongest positive correlation with the bacterial infection target. Enoxaparin and anticoagulants also showed significant associations with the bacterial infection. Additionally, the initial fever finding and basophils exhibited moderate positive correlations. Conversely, features such as the mAb of tocilizumab and the susceptibility to amikacin showed weak negative correlations with the target. Other antibiotic susceptibility variables, including susceptibility to cefepime and ciprofloxacin, had minimal or near-zero correlations, indicating a limited direct association with the outcome. The predictive performance of the logistic regression models for both outcomes is summarized in Table 5. The mortality outcome model achieved robust performance, with the area under the ROC curve (AUROC) of 0.90 (95% CI: 0.85–0.95) in the training set and 0.88 (95% CI: 0.83–0.93) in the test set, indicating strong discriminative ability. The bacterial infection outcome model demonstrated even higher predictive power, with AUROC exceeding 0.96 in both training and test sets, accompanied by perfect sensitivity (1.00) in both cases. Table 6 reports a comparative summary of the top predictors identified by four variable-importance methods—mutual information, LASSO coefficients, logistic regression odds ratios, and mean SHAP values—highlighting both overlaps and divergences in feature ranking. Variables such as zinc, vitamin C, enoxaparin, and neutrophils consistently emerged as key predictors across multiple methods, reinforcing their clinical relevance in predicting mortality among patients with CAP. Meanwhile, other variables such as meropenem, pH, and platelet count showed importance only in selected methods, underscoring the need for careful interpretation of variable-importance results depending on the statistical approach. Table 7 presents forest plot data for key predictors of both investigated outcomes in this study. Variables such as ICU stay, pH, WBC, and platelets exhibited significant associations with mortality risk, with odds ratios ranging from 0.6 (for pH) to 2.5 (for ICU stay). For the bacterial infection outcome, predictors such as lymphocytes, HCO_3_, WBC, and neutrophils had odds ratios between 0.8 and 2.5. These forest plots provide valuable insights into the relative importance and accuracy of these predictors, supporting their potential utility in clinical decision-making and model interpretability.

**Table 7:** Forest plot data

**Variable**	**Odds ratio**	**95% CI**	***P* value**
*Forest plot data for mortality outcome*			
ICU stay	2.5	1.5–4.1	0.001
pH	0.6	0.4–0.9	0.025
WBC	1.8	1.2–2.7	0.005
Platelets	1.7	1.1–2.6	0.010
Meropenem	2.0	1.3–3.1	0.003
*Forest plot data for bacterial infection outcome*			
Lymphocytes	1.4	1.0–2.0	0.045
WBC	2.1	1.5–3.0	0.002
MCV	1.3	0.9–1.9	0.060*
Neutrophils	2.5	1.8–3.4	0.001
Basophils	1.6	1.1–2.4	0.010

*Despite being statistically insignificant, it is kept in the table due to its close to the significant limit. Abbreviations: ICU: Intensive care unit; WBC: White blood cell count; MCV: Mean corpuscular volume.

## Discussion

Authors should discuss the results and interpret them in the context of previous studies and working hypotheses. The findings and their implications should be explored in the broadest context possible. Additionally, the limitations of the study and potential future research directions should be addressed. The primary aim of this study was to develop, validate, and implement ML models specifically designed to predict the etiology and mortality of CAP. Among the 251 patients included, 215 (85.7%) had bacterial CAP, with most surviving (67.3%). Treatment regimens were extensive, with high utilization of antibiotics and anticoagulants. The severity of pneumonia varied across patients.

### Predicting mortality of CAP

In the Feature Correlation Analysis Regarding Mortality step, the results showed that zinc, vitamin C, enoxaparin and insulin bolus had high correlation with CAP in hospital survival rate.

Regarding zinc supplement; The clinical studies show same results as increasing zinc intake will decrease the mortality or increase the survival rate. One study result was: Zinc deficiency, common in developing countries, is linked to increased CAP morbidity and mortality. Zinc levels were lower in older patients, those with high CURB-65 scores, and smokers [32].

The feature correlation also showed high correlation for vitamin C supplement and in hospital survival rate, the clinical findings also agree with. In one systematic review showed that vitamin C supplementation had potential benefits on CAP management [33].

These nutrients are often studied for their potential benefits in supporting the immune system and improving outcomes in respiratory infections [34]. in our study they were taken as a chronic treatment emphasizing their role as immunity supporters.

The feature correlation analysis also showed a high correlation between enoxaparin and in-hospital survival rate, this result is alongside the clinical findings. One study conducted, they found that Enoxaparin is associated with lower rates of mortality. Enoxaparin is often used as a preventative measure in hospitalized CAP patients to reduce the risk of venous thromboembolism, a serious complication that can worsen outcomes the pateints [35].

About insulin bolus treatment; Severe infections like CAP trigger a stress response, leading to increased levels of cortisol, catecholamines, and inflammatory cytokines [36]. These factors contribute to insulin resistance and hyperglycemia, even in non-diabetic patients. In diabetic patients, CAP can cause poor glycemic control, increasing the risk of complications. Persistent hyperglycemia weakens the immune response, impairing neutrophil function and increasing infection severity [37]. Also high blood sugar levels are linked to higher mortality rates, prolonged hospital stays, and increased risk of complications such as sepsis and respiratory failure [38].

Insulin therapy in adult patients hospitalized for critical illness, excluding hyperglycemic crises, may reduce mortality in certain patient groups [39]. This effect also extends to insulin bolus treatment, which has been shown to decrease both mortality rates and LOS [40]. The next step in ML modeling for CAP mortality predictors was Mutual Information analysis. The analysis quantified the dependency between each feature and the target (mortality), providing a ranking of the most informative predictors. Neutrophil count, ALT, MCV, HGB, and platelet count emerged as the top contributors. These findings emphasize the importance of these variables, as they are often indicative of systemic inflammation. The High neutrophil/lymphocyte ratio (NLR) and neutrophil count percentage (NCP) are reliable predictors of mortality [41]. Overactive neutrophils release pro-inflammatory cytokines that can cause tissue damage and thrombosis [42]. Regarding ALT levels, one study demonstrated that circulating liver function biomarkers exhibit diverse nonlinear correlations with mortality [43]. CAP triggers a systemic inflammatory response, which can elevate ALT levels due to liver stress. In the case of MCV, one study found that large MCV was associated with all-cause mortality, cardiovascular disease mortality, and infection-associated mortality [44]. Elevated MCV may contribute to venous thromboembolic disease through increased hematocrit, which promotes platelet margination, or by increasing blood viscosity, which reduces flow in large vessels and predisposes to clot formation. This imbalance in blood rheology can lead to thrombosis [45]. Patients with abnormal platelet counts—either thrombocytopenia (19%) or thrombocytosis (28%)—had longer hospital stays, a higher need for ICU admission, increased use of mechanical ventilation (both invasive and noninvasive), and a higher 30-day mortality rate [46]. This is consistent with our model’s results.

An increase in HGB level predicts an increased in mortality, ICU hospitalization, and extrapulmonary complications in COVID-19 patients [47]. Elevated HGB increases blood viscosity, slowing circulation and raising the risk of thrombosis, stroke, and cardiovascular disease [48]. These clinical findings align the result regarding the mutual information analysis.

Feature selection through Lasso regression further refined list of predictors by identifying the magnitude and direction of their contributions to the mortality target. Features such as Meropenem, ABGs, PCO_2,_ and platelet count demonstrated strong positive coefficients, suggesting their importance in predicting the mortality target. Conversely, features such as location and cardiovascular disease were negatively associated with the mortality outcome.

Regarding meropenem empirical treatment (in our data records meropenem used as empirical treatment). Empiric meropenem-based regimen appeared to be associated with lower mortality [49]. But in recent study they found that meropenem increases mortality rate; 50 patients (14.12%) experienced treatment failure, and ICU mortality was 48.02% (120 patients). Predictors of meropenem failure included a higher APACHE (acute physiology and chronic health evaluation) score and shorter treatment duration. Predictors of mortality were high APACHE and SOFA scores, initiation of antibiotics more than 72 h after sepsis onset, shorter treatment duration, and renal dose adjustments of meropenem [50]. The last study align with our model result.

Regarding ABGs, one study related to COVID-19 mortality, ABGs was found as a predictor of mortality in COVID pneumonia patients initiated on noninvasive mechanical ventilation [51].

Regarding the positive relationship with PCO_2_, clinical findings have same result with higher PCO_2_ being associated with worse survival [52]. Platelet count results also agree with the clinical findings. Findings among patients with mild thrombocytosis suggested that high-normal platelet count is associated with the occurrence of thrombotic events [53].

We had negative or inverse relationships (CVDs, COVID 19 and location) that may need more focusing on features or model selection as these findings should have positive impacts. The patient with severe CAP needs monitoring of an ICU where, if necessary, they can receive specialized support connected to a mechanical ventilator and/or hemodynamic support [54]. Also, patients can get pneumonia when infected with SARS-COV_2_. The virus that causes COVID-19 can infect the lungs, causing pneumonia [55]. Finally CAP is a significant risk factor for all major cardiovascular disease events, including acute coronary syndrome, stroke, and mortality [56]. These clinical findings (CVDs, COVID-19 and location) have opposite relationship regarding our modeling results, so farther processing and handling must be needed regarding lasso regression model. Regarding pH, one study showed metabolic acidosis (low pH) is associated with higher mortality in ICU [57]. This inverse relationship agreed with our model results.

The last step used was Logistic regression. It provided an additional perspective on variable significance. Variables such as pH, ICU_LOS, LOS, age, PCO_2_, O_2_ saturation, and diastolic blood pressure were identified as the most impactful predictors of the mortality target outcome. Regarding pH, inverse relationship emphasized by clinical finding [57].

Regarding LOS as mentioned earlier; the patient with severe CAP needs monitoring of an ICU where, if necessary, they can receive specialized support connected to a mechanical ventilator and / or hemodynamic support [54]. PSI, WBC, PCO_2_, HCO_3_, ABGs and diastolic blood pressure have positive relationship alongside the clinical finding [52, 58–60]. Among outpatients with pneumonia, oxygen saturations <90% were associated with increased morbidity and mortality [61]. This agree with our model result.

Our aims of this study to make rapid evaluation about patient condition at the admission time; by looking for the results obtained (zinc, vitamin C supplements, Enoxapirin, Neutrophils count, platelet count, ALT, MCV, ABGs, PCO_2,_ HGB, pH, WBC, HCO_3_ and O_2_ saturation) are main predictors for mortality and these results can be obtained rapidly at the admission time [62]. There are some predictors have opposite clinical findings results such as CVDs and COVID-19 these findings indicate that we must do further investigation and choosing for models and features selection.

### Predicting the etiology of CAP

A heatmap was generated to assess the correlation between clinical variables and the target outcome (infection with bacteria). Enoxaparin, along with other anti-coagulant treatments, exhibited the strongest positive correlations with bacterial infection. However, this correlation suggests negative clinical outcomes. Severe bacterial pneumonia and sepsis trigger coagulation activation both locally in the lungs and systemically, leading to tissue injury and reduced survival. This has prompted extensive investigation into therapies that modulate inflammation caused by coagulation activation. Various anticoagulant strategies have been explored for bacterial pneumonia, sepsis, and acute respiratory distress [63]. Notably, Vitamin C supplementation also showed a strong correlation with Zinc in relation to bacterial CAP. These results suggest opposing clinical outcomes. A recent study demonstrated that higher doses of vitamin C and zinc resulted in greater inhibition of Klebsiella pneumoniae biofilm formation, indicating their potential as alternative agents to combat biofilm-associated antibiotic resistance [64]. Logistic regression analysis to evaluate feature importance revealed that lymphocytes were the most influential predictor. This finding contrasts with some clinical research [65, 66]. Serum bicarbonate levels (HCO_3_) showed similar results to clinical studies, where elevated serum bicarbonate levels have been observed in patients with bacterial pneumonia [67]. Severe bacterial lung infections, such as pneumonia, can cause respiratory acidosis due to poor gas exchange and carbon dioxide retention. Regarding WBCs, typical bacterial CAP was associated with WBC counts ≥15,000/mL [65]. This aligns with our results. In terms of BUN, one study on bacterial urinary tract infections observed a decrease in BUN levels [68], which matches our model’s results. The most important clinical predictor for bacterial infection is neutrophil count, as neutrophils typically increase during bacterial infections. However, our model showed an opposite result [65, 66]. SHAP values were employed to interpret model predictions, Antibiotics, Basophils, and initial finding fever emerged as key variables influencing the model’s output.

Bacterial CAP needs antibiotic treatment [69] and obtaining result opposite for this (antibiotics intake increases bacterial infection) has no clinical meaning.

About basophils. A study performed on mice found that basophils can enhance the innate immune response against bacterial infection and help prevent sepsis [70].

While fever is a key feature of both bacterial and viral CAP, its presence and characteristics alone are not sufficient to differentiate the two. A combination of clinical, laboratory, and imaging findings is necessary for accurate diagnosis. The symptoms of bacterial pneumonia can develop gradually or suddenly. Fever may rise as high as a dangerous 105 degrees F, with profuse sweating and rapidly increased breathing and pulse rate [71].

Lastly, The correlation coefficients between selected features and the target variable. The most clinical predictors are initial fever and Basophils that are alongside with clinical finding [70, 71].

In summary, models showed (HCO_3_, BUN, WBC, and initial fever) are main predictors for bacterial CAP. Also showed opposite clinical results with lymphocyte count. This indicates we need further models and features selection.

The small sample size (*n* ═ 251) may limit the generalizability and robustness of our findings. As part of future work, we aim to scale up the study by collaborating with additional clinical sites to collect a larger, multi-center dataset with consistent data quality standards. This expansion will enable more rigorous model training, external validation, and improved confidence in the predictive performance of the ML models.

The observational nature of the dataset introduces potential confounding variables that may not have been fully accounted for in the models. Certain predictors, such as medication usage (e.g., enoxaparin or insulin bolus), may reflect treatment decisions based on illness severity rather than causal relationships. Additionally, imbalances in the distribution of bacterial vs non-bacterial CAP, or survivor vs non-survivor groups, could introduce class bias into model training, influencing performance and feature interpretation.

Different ML models have inherent assumptions and biases that can affect variable selection and interpretation. For instance, LASSO regression is effective for feature reduction but limited in capturing non-linear interactions. Random forest models handle non-linearity but may overemphasize variables with high cardinality or variance. SHAP values, while providing detailed interpretability, may diverge from classical regression coefficients and can be misinterpreted without sufficient contextual knowledge. Inconsistencies between SHAP results and established clinical findings (e.g., regarding antibiotics or lymphocyte counts) further underscore the need for domain-aware interpretation.

Although the study focuses on early admission features, not all patients had complete laboratory or radiological data available at standardized time points. Missing values may have necessitated imputation or led to the exclusion of potentially informative features. Furthermore, variables like medication use (e.g., antibiotics or vitamins) may have been recorded without precise timing relative to symptom onset or diagnosis, complicating causal inference.

## Conclusion

This study explored key predictors of mortality and bacterial etiology in CAP, using ML models to analyze various clinical features. Key findings include the association of zinc and vitamin C supplements, as well as enoxaparin, with increased survival rates. Neutrophil count, ALT, MCV, HGB, and platelet count were linked to increased mortality, while insulin bolus therapy was shown to reduce mortality, emphasizing the importance of glycemic control in CAP patients. However, some variables, such as lymphocyte count, COVID-19, and cardiovascular disease, yielded conflicting results, suggesting the need for further investigation. Regarding bacterial etiology, features like HCO_3_, BUN, WBC, and initial fever were significant predictors, although discrepancies in lymphocyte count highlight the need for model refinement. Overall, rapid evaluation of clinical variables at admission is crucial for predicting mortality and bacterial infections in CAP, but further validation and model adjustments are needed for more accurate outcomes.
